# GENERAL AND GENDER-SPECIFIC DYNAMICS DETERMINING THE HEALTH STATUS OF WORKING INFORMAL CAREGIVERS

**DOI:** 10.1093/geroni/igz038.2699

**Published:** 2019-11-08

**Authors:** Abby Schwartz, Leanne J Clark-Shirley, G Rainville

**Affiliations:** 1 East Carolina University, School of Social Work, Greenville, North Carolina, United States; 2 AARP, Washington, District of Columbia, United States

## Abstract

As the gender divide among family caregivers closes, it is important to understand whether and how men and women differently experience caregiving. For example, literature suggests that employed family caregivers experience burden and health outcomes differently than unemployed caregivers, but less is known about how these factors affect men and women differently. Using data from Caregiving in the U.S., 2015 (source: AARP and NAC), this study reliably modelled the effect of multiple threats to good health within the caregiving role (e.g. physical, financial, and emotional strain). In the analysis, several moderated relationships were observed using data from 816 working caregivers. In the full sample, the relationship between objective caregiving burden (hours of care and counts of ADLs/IADLs) and self-reported health status was altered by financial strain. In the high burden condition, relatively poor health was progressively related to increasing levels of financial strain controlling for traditional covariates. In separate analyses for males and females, this moderated relationship was discovered to be limited to female caregivers. Physical and emotional strain did not moderate the relationship between burden and health. Several covariates related to employment conditions (e.g., caregiver-friendly workplace policies) behaved differently across models and are presented and discussed in relation to financial strain as a determinant of caregiver health. These findings shed light on gender-based differences in caregiver outcomes, and suggest that interventions aimed at assessing and improving caregiver health should account for the financial strain experienced particularly by women.

